# Lithium-Based Upconversion Nanoparticles for High Performance Perovskite Solar Cells

**DOI:** 10.3390/nano11112909

**Published:** 2021-10-30

**Authors:** Masfer Alkahtani, Anas Ali Almuqhim, Hussam Qasem, Najla Alsofyani, Anfal Alfahd, Sultan M. Alenzi, Abdulaziz Aljuwayr, Yahya A. Alzahrani, Abdurahman Al-Badri, Mohammad Hayal Alotaibi, Abdulaziz Bagabas, Abdulaziz N. AlHazaa, Philip R. Hemmer

**Affiliations:** 1 National Center for Renewable Energy, King Abdulaziz City for Science and Technology (KACST), Riyadh 11442, Saudi Arabia; amukhem@kacst.edu.sa (A.A.A.); hqasem@kacst.edu.sa (H.Q.); nalsofyani@kacst.edu.sa (N.A.); aalfahd@kacst.edu.sa (A.A.); jwr881@gmail.com (A.A.); 2 Institute for Quantum Science and Engineering, Texas A&M University, College Station, TX 77843, USA; prhemmer@exchange.tamu.edu; 3 National Center for Nanotechnology and Semiconductors, King Abdulaziz City for Science and Technology (KACST), Riyadh 11442, Saudi Arabia; sultan0064@gmail.com (S.M.A.); yalzhrani@kacst.edu.sa (Y.A.A.); aalbadri@kacst.edu.sa (A.A.-B.); 4 National Petrochemical Technology Center (NPTC), Materials Science Research Institute (MSRI), King Abdulaziz City for Science and Technology (KACST), Riyadh 11442, Saudi Arabia; mhhalotaibi@kacst.edu.sa (M.H.A.); abagabas@hotmail.com (A.B.); 5 Research Chair for Tribology, Surface, and Interface Sciences (TSIS), Department of Physics and Astronomy, College of Science, King Saud University, Riyadh 11451, Saudi Arabia; aalhazaa@ksu.edu.sa; 6 King Abdullah Institute for Nanotechnology, King Saud University, Riyadh 11451, Saudi Arabia; 7 Department of Electrical and Computer Engineering, Texas A&M University, College Station, TX 77843, USA; 8 FRC Kazan Scientific Center of RAS, Zavoisky Physical-Technical Institute, Sibirsky Tract, 10/7, 420029 Kazan, Russia

**Keywords:** perovskite solar cell, upconversion nanoparticles, lithium, efficiency

## Abstract

In this work, we report an easy, efficient method to synthesize high quality lithium-based upconversion nanoparticles (UCNPs) which combine two promising materials (UCNPs and lithium ions) known to enhance the photovoltaic performance of perovskite solar cells (PSCs). Incorporating the synthesized YLiF_4_:Yb,Er nanoparticles into the mesoporous layer of the PSCs cells, at a certain doping level, demonstrated a higher power conversion efficiency (PCE) of 19%, additional photocurrent, and a better fill factor (FF) of 82% in comparison to undoped PSCs (PCE = ~16.5%; FF = 71%). The reported results open a new avenue toward efficient PSCs for renewable energy applications.

## 1. Introduction

Over the decades, renewable energy has attracted special attention and has been considered to be the best alternative to conventional energy sources such as oil and natural gas [1,2,3]. Among the renewable energies, solar energy is still the most abundant, environmentally friendly energy form to ensure the world’s continued prosperity. Crystalline silicon-based photovoltaic (PV) cells are the most used solar cells to convert sunlight into electricity, providing clean energy for many interesting applications with moderately high operating efficiencies between 20% and 22% [3,4,5]. The Si-based PVs are a mature, highly optimized technology with little margin for enhancing their efficiency. However, purification, reduction, and crystallization of pure silicon from sand require sophisticated industrial processing, which is highly energy demanding and causes undesirable pollution to the environment [4,6]. In addition, there are much more efficient solar cells, for example, gallium arsenide (GaAs)-based solar cells, but they are quite expensive and suffer degradation [7]. Also, organic photovoltaics (OPVs) have recently attracted considerable attention but are still limited by low stability and low strength in comparison to inorganics solar cells [8,9].

As an alternative, perovskite-based solar cells (PSCs) have made impressive, unprecedented advances with power conversion efficiencies reaching 25.2% in the past ten years [10,11,12] due to the extraordinary characteristics of perovskite materials, such as a long charge carrier diffusion length [13,14,15], a high absorption coefficient in the visible band of the solar spectrum [13,16], and simple manufacturing processes [13,17]. In PSCs, perovskite is the light-harvesting active layer, which consists of a perovskite-structured compound in ABX_3_ (hybrid organic–inorganic) composition. In this composition, an organic cation A is usually made of promising materials such as methylammonium (MA) or formamidinium (FA) [18,19], while the [BX3]^−^ anion is usually made of inorganic materials based on lead or tin [20,21], where the halide X ion is Br or I.

To enhance the photovoltaic performance of PSCs, efforts have been made to introduce additive light-harvesting materials to the perovskite to fully utilize the sunlight, and therefore, to improve power conversion efficiency [10,22,23,24,25,26,27]. Lanthanide (rare-earth) ion-doped upconversion nanoparticles (UCNPs) have shown great potential as spectral converters to harvest near-infrared (NIR) solar photons from sunlight and convert them to absorbable visible light photons by the perovskite light-harvesting active layer [13,28,29,30]. UCNPs have been incorporated into different layers of the PSCs to improve their photovoltaics performance and PCE. UCNPs in core–shell architecture were doped into the mesoporous layer of PSCs [31,32,33], and sodium fluoride UCNPs (NaYF_4_:Yb,Er) were incorporated as a scaffold of H_3_CNH_3_[PbI_3_] crystals [34]. Moreover, β-NaYF_4_:Yb,Er nanoparticles were added as an interface layer between perovskite and the spiro layer [35]. Furthermore, Li(Gd, Y)F_4_:Yb,Er UCNPs nanocrystals were added into the hole transport layer of PSCs [36].

Apart from the UCNPs’ function as a spectral converter, UCNPs also act as a scattering layer, which can increase the light path [37]. They can also help the formation of large perovskite grains with fewer defects [27], which further enhance the photovoltaic performance of PSCs. As a result, PSCs with rare-earth doped UCNPs have achieved a higher power conversion efficiency (PCE) in comparison to undoped PSCs cells.

In addition to enhancing absorption, effort has been made to enhance the electron/hole transport layer by doping with various materials to give superior electrical properties [10,13,25,26]. It was demonstrated that Li-doping in the mesoporous TiO_2_ layer of PSCs enabled faster electron transport within the TiO_2_ electrodes, and therefore, it achieved substantially higher photovoltaic performance and enhanced PCE with negligible hysteresis behavior in comparison to the undoped PSCs [10].

Engineering a hybrid system, containing both of these promising materials, UCNPs and lithium, is of a special interest for efficient PSCs. Recently, this idea has been explored by doping lithium and lanthanide ions directly into TiO_2_ crystals, which are then put into the PSCs. However, so far the photovoltaic performance of PSCs, fabricated with a Yb–Er–Li tri-doped TiO_2_ hybrid system, have shown a relatively low PCE of 16% [29] in comparison to PSCs fabricated with fluoride-based UCNPs [13] and lithium-doped TiO_2_ as reported in [10]. This low performance could be attributed to low quantum efficiency of UCNPs, doped in oxide crystals, due to their relatively high phonon energy [38,39]. In contrast, fluoride crystals have a lower phonon energy, and give relatively higher quantum efficiency because the low phonon energy slows the decay of the intermediate state by requiring multiphonon relaxations [40,41].

In this work, we report an easy, efficient way to synthesize lanthanide ions doped lithium-fluoride-based crystals (YLiF_4_) and incorporate them into solar cells. YLiF_4_ crystals are promising materials for creating active optical environment with high light output when they are exposed to UV and visible light irradiations [42]. It was shown that active optical defects in YLiF_4_ crystals were responsible for several absorption bands in the UV and visible regions, which are important for perovskite cells [42,43]. The synthesized hybrid system showed ultrabright and small (13 nm) YLiF_4_: Yb,Er upconversion nanoparticles. The photovoltaics performance of the PSCs, fabricated with the synthesized UCNPs, was evaluated, and demonstrated an increase in PCE of 19% in comparison to the undoped PSCs cells. These results suggest an easy, efficient strategy to combine these promising materials into a hybrid system to enhance the efficiency of PSCs.

## 2. Materials and Methods

### 2.1. Preparation of LiYF_4_:Yb,Er (18/2%) Nanocrystals

An amount of 1.0 mmol of LnCl3 (Ln = Y (80.0 wt.%), Yb (18.0 wt.%), and Er (2.0 wt.%)) was placed into a 100 mL three-neck flask containing 10.5 mL of oleic acid and 10.5 mL of 1-octadecene. This mixture was then heated to 150 °C for 40 min under argon flow to obtain a clear yellow solution. Afterwards, the solution was cooled down to 50 °C, and a mixture of 5.0 mL of methanol solution with 2.5 mmol of LiOH.H_2_O and 10.0 mL of methanol solution with 4.0 mmol of NH_4_F was slowly injected and was maintained at 50 °C for 40 min under a vigorous stirring. The solution was then slowly heated to 150 °C and was kept for 20 min to remove the methanol and residual water. The reaction mixture was then heated to 280 °C for 1.5 h under argon flow. After the reaction was complete, the mixture was cooled down to room temperature, and the synthesized LiYF_4_:Yb,Er UCNPs were collected, washed three times with ethanol, and re-dispersed in 10 mL of chloroform.

### 2.2. Preparation of Ligand-Free Ln-UCNPs for the Solar Cell Experiment

A volume of 1.0 mL of the oleate-capped LiYF_4_:Yb,Er UCNPs nanoparticles was dispersed in 40 mL of acidic ethanol solution (pH = 1) adjusted by concentrated hydrochloric acid. The solution was then sonicated for 1 h to remove the oleate ligands. Afterwards, the nanoparticles were collected via centrifugation at 14,500 rpm for 30 min and were washed three times with ethanol/water (1:1 *v*/*v*). The oleate-free Ln-UCNPs were then re-dispersed in absolute ethanol for further use.

### 2.3. Preparation of Mesoporous Layer

The mesoporous layer of the fabricated PSCs was prepared by mixing the synthesized LiYF_4_:Yb,Er (various sizes in the range of 10–15 nm) with commercial TiO_2_ paste (particle size of 30 nm) (Dyesol 30NRT, Dyesol). For this purpose, an amount of 1 mmol of ligand-free Ln-UCNPs was dissolved into 1.0 mL of absolute ethanol. The solution was then mixed with the commercial TiO_2_ paste corresponding to the desired different mixing ratios (UCNPs:TiO_2_ = v(UNCPs) × 100/v(TiO_2_), *v/v*, x = 0, 15, 30, 40, and 50). For clarity, the samples were named after this mixing ratio as follows: pristine (0% of UCNPs), device-15% UCNPs, device-30% UCNPs, device-40% UCNPs, and device-50% UCNPs. The mixtures were diluted in different amounts of absolute ethanol to keep the concentration of 0.1 gmL^−1^, were ultrasonicated, and then were kept under overnight stirring.

### 2.4. Perovskite Solar Cells Devices Fabrication

First, fluorine-doped tin oxide (FTO) glass substrates were cleaned by sonication in soap (Hellmanex2.0) for 30 min, in de-ionized water for 5 min, ethanol for 15 min, and acetone for 15 min, respectively. After cleaning, the FTO substrates were treated with UV-ozone for 30 min.

Second, a thin compact TiO_2_ layer for all FTO substrates was prepared by spin-coating of a solution containing 0.6 mL of titanium isopropoxide, 0.4 mL of acetylacetonate, and 9.0 mL of ethanol onto the substrates at 2500 rpm for 30 s, and then was annealed at 450 °C for 30 min. After this step was complete, the mesoporous layer (thickness of 150–200 nm) of the UCNPs was then introduced on the top of the compact layer by spin coating the mixed paste/UCNPs at 5000 rpm for 30 s and then was annealed at 500 °C for 30 min.

Third, to prepare a perovskite precursor solution, a solution of materials (18.84 mg of methylammonium bromide, 247.2 mg of formamidine bromide, 722.22 mg of lead(II) iodide, 62.04 mg of lead(II) bromide, 21.84 mg of cesium iodide, 960 µL of dimethylformamide (DMF), and 240 µL of dimethyl sulfoxide (DMSO)) was mixed and heated to 80 °C for 15 min to ensure homogeneity to achieve the triple cation composition. Afterwards, 50 µL of the precursor solution was spin-coated at two different rpm speeds: at 1000 rpm for 10 s and then at 6000 rpm for 30 s, respectively. To remove residual DMSO and DMF in the precursor films, 200 μL of chlorobenzene was poured on the substrates for 15 s and the substrates were then annealed at 100 °C for 45 min on a hotplate to form crystalline triple cation perovskite layers.

Fourth, a hole transfer layer (HT) was subsequently deposited on top of the triple cation perovskite layers by the spin-coating of a solution of N2,N2,N2′,N2′,N7,N7,N7′,N7′-octakis(4-methoxyphenyl)-9,9′-spirobi [9H-fluorene]-2,2′,7,7′-tetramine (spiro-MeOTAD) at 4000 rpm for 20 s. After that, an 80 nm thick gold layer was thermally deposited on the top of the spiro-MeOTAD layers under high vacuum by using a special shadow mask. Finally, the fabricated PSCs devices with an active area of 0.1 cm^2^ (0.25 × 0.4 cm^2^) were prepared for the photovoltaic performance measurements.

## 3. Results and Discussion

**Figure 1:**
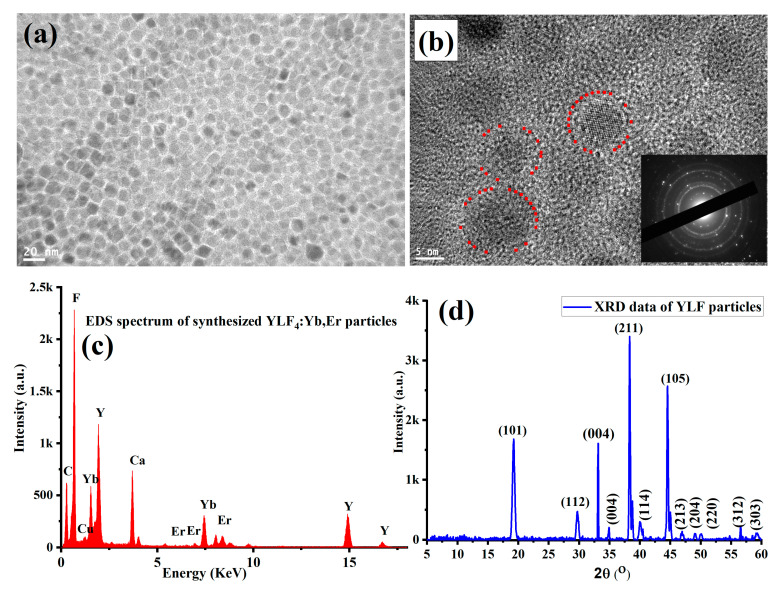
Characterizations of the synthesized YLiF_4_:Yb,Er UCNPs. (**a**) Low magnification TEM image of the synthesized UCNPs showing ultrasmall (less than 15 nm) and well-dispersed nanoparticles. (**b**) High magnification image of the synthesized UCNPs showing crystalline UCNPs. (**c**) Energy-dispersive X-ray (EDX) spectrum for the elemental analysis of the synthesized UCNPs. (**d**) XRD pattern of UCNPs with relatively sharp peaks for crystalline, high-quality particles.

The lithium-fluoride-based UCNPs (YLiF_4_:Yb,Er) were synthesized by following a solvent thermal protocol reported in [44] and detailed in the Material and Methods section. To visualize the size and morphology of the synthesized UCNPs, a few drops of the sample were placed on a carbon-coated copper grid of a transmission electron microscope (TEM). Figure 1a,b shows low and high magnification TEM images of ultrasmall, well-dispersed, and crystalline UCNPs particles with an average size of 13 nm. The qualitative composition of the synthesized YLiF_4_:Yb,Er UCNPs was confirmed by the energy-dispersive X-ray (EDX) spectrum, as illustrated in Figure 1c. The X-ray diffraction pattern (XRD) of the synthesized UCNPs in Figure 1d revealed relatively sharp peaks, indicating crystalline, high-quality UCNPs.

**Figure 2:**
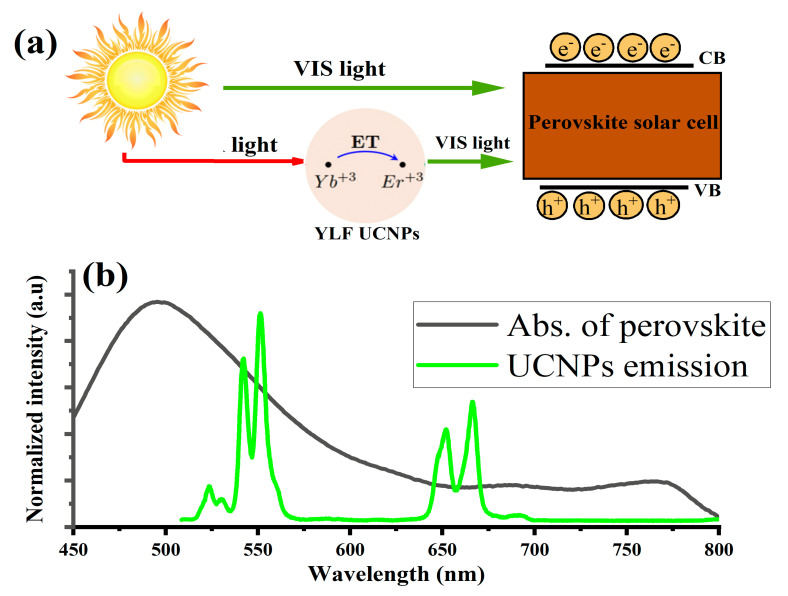
(**a**) Schematic illustration of how the synthesized YLiF_4_:Yb,Er UCNPs absorb and convert near-infrared photons from the sunlight to visible light within the absorption band of the light-harvesting layer of the PSC. (**b**) PSCs absorption band overlaps with UCNPs green emission peaked at 550 nm and red emission at 650 nm, respectively.

The UCNPs were introduced into the PSCs device in the mesoporous layer at different mixing ratios with TiO_2_ nanoparticles, as detailed in Section 2. The goal was to convert the NIR bands of the solar spectrum into a visible light, which can be harvested by the perovskite active layer, as illustrated in Figure 2a. To fully utilize this strategy, it was important to carefully choose an upconverting ion of the rare earth elements with perfect matching emission to the perovskite light-harvesting absorption band. One of the best candidates was erbium (Er^+3^), which emits an intense green and red light within the visible range of the perovskite active absorption, as illustrated in Figure 2b.

In YLiF_4_:Yb,Er combination, the doping of Yb^3+^ and Er^3+^ ions in the YLiF_4_ host lattice will substitute the Y^3+^ site due to their identical charge of (+3). The optical process in this combination is based on the sequential absorption of two photons. The ytterbium (Yb^+3^) ion acts as a sensitizer for absorbing and transferring the NIR photon energy to the erbium ion (Er^+3^) in two steps: first to its intermediate and then to its excited states, respectively. Afterwards, the highly excited states of the erbium ion (Er^+3^) relax to lower excited states through multiphonon relaxations, followed by radiative emission in the visible range of 500–700 nm. The visible emission consists of two bands in the green region and one band in the red region, which are the characteristic transitions of the Er^3+^ ion according to the following optical transitions: ^2^H_11/2_ → ^4^I_15/2_ (525nm), ^4^S_3/2_ → ^4^I_15/2_ (555 nm) and ^4^F_9/2_ → ^4^I_15/2_ (654 nm). The crystal field splitting of the Er^3+^ emission lines in the optical emission spectrum of the synthesized YLiF_4_:Yb,Er is different from that of the commonly used β-NaYF_4_:Yb,Er phosphors. This difference is due to the point symmetry of the rare earth ion sites in the inverse scheelite (CaWO_4_) structure of YLiF_4_:Yb,Er UCNPs, with S_4_ instead of C_3h_ symmetry [45,46,47].

**Figure 3:**
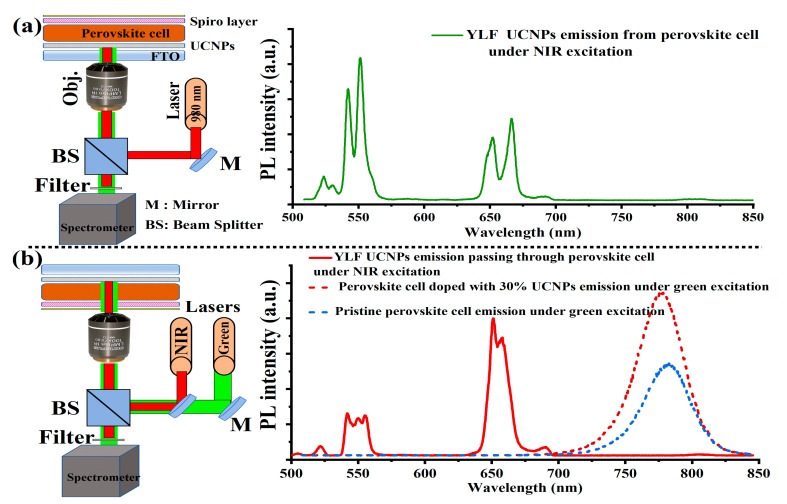
(**a**) Schematic illustration of a home-made confocal microscope designed and equipped with 980 nm laser for photoluminescence (PL) measurement of the PSC layers on FTO/UCNPs/perovskite/spiro layer. Optical spectrum in Figure 3a shows UCNPs emission spectrum measured directly from the UCNPs layer without passing through perovskite layer. (**b**) Illustration of the same optical setup, equipped with green and NIR lasers for PL measurement of the UCNPs and perovskite layers within the PSCs devices layers. The optical spectra in Figure 3b show how the UCNPs emission collected through the perovskite layer and was strongly absorbed, especially at the green band at 550 nm and partially absorbed at 650 nm band. Figure 3b also shows the PL spectra of perovskite films with and without UCNPs doping within the mesoporous layer.

To investigate the absorption of the upconverted light emission from the UCNPs by the perovskite light-harvesting layer, we fabricated a PSCs device without gold electrodes to allow for light transmission. In this device, UCNPs were mixed with TiO_2_ nanoparticles in a mesoporous layer at a mixing ratio of UCNPs:TiO_2_ (30%:70%, volume ratio), as this mixing ratio was reported [13] to give the highest PSCs photovoltaic performance. To optically characterize the fabricated layers, we designed and built a custom-made confocal laser-scanning microscope, equipped with continuous wave (CW) 532 nm (green) and 980 nm (NIR) lasers, an optical spectrometer, and a single-photon counter, as illustrated in Figure 3a,b. The PSCs device was placed on the optical setup and was irradiated with 980 nm laser on both sides. In the case of Figure 3a, the UCNPs emission was collected by the optical spectrometer without absorption. In contrast, in the case of Figure 3b, the optical emission of the UCNPs was detected after passing through the perovskite layer. As seen in Figure 3b, the green wavelength of the UCNPs emission was strongly absorbed by the perovskite layer during penetration while the red emission, peaked at 650 nm, remained almost unchanged. The reduction percentage of the green and red emission intensities of the upconverted light, absorbed by the perovskite layer, was estimated to be 35% and 41%, respectively. The strong green absorption by the perovskite layer was due to a good overlap between the UCNPs green emission and the maximum absorption band of the perovskite layer. This absorption of upconverted light suggested that UCNPs in the mesoporous layer should improve PCE. The optical emission from the perovskite material with and without UCNPs doping was investigated under green excitation. The photoluminescence of the perovskite film peaked at 780 nm with UCNPs-30% doped within the mesoporous layer, was higher than that of the pristine film, as shown in Figure 3(b). This observation could be attributed to the reduction of grain boundaries by UCNPs addition [13], a decrease in the non-radiative recombination, and the defect trap states [13].

To investigate the photovoltaic performance of the PSCs with and without UCNPs, we fabricated several devices with different weight ratios of UCNPs/TiO_2_ in the mesoporous layer. The fabricated devices were named as follows: pristine-0%UCNPs, device-15% UCNPs, device-30% UCNPs, device-40% UCNPs, and device-50% UCNPs. Materials and methods used in devices fabrication are detailed in Section 2. We experimentally performed photocurrent density-voltage curves (J-V) of the fabricated devices under 1-sun illumination at AM 1.5 G to test the photovoltaic performance of the fabricated PSCs.

**Figure 4:**
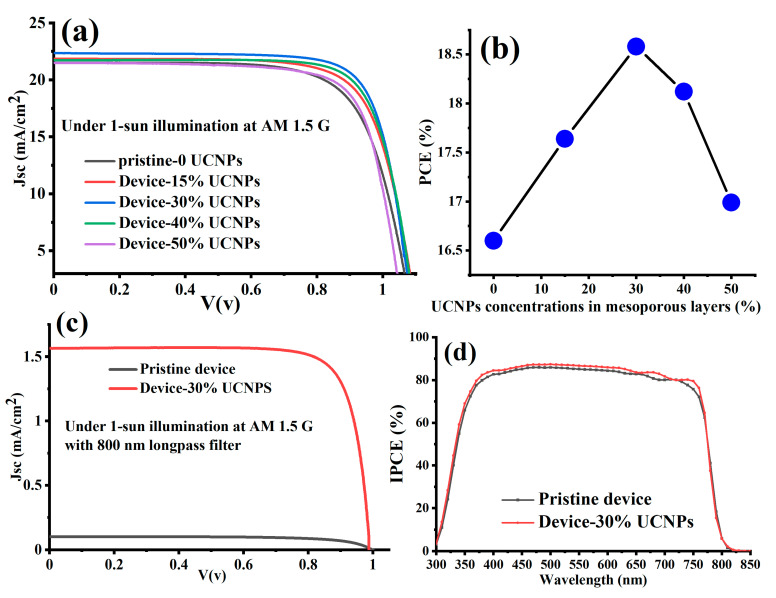
(**a**) J-V characteristic curves measured under AM 1.5 G for fabricated PSCs with and without UCNPs amounts integrated within the mesoporous layers. (**b**) PCE of the fabricated PSCs as a function of the UCNPs amounts integrated within the mesoporous layers. (**c**) J-V characteristics measured under NIR irradiation with 800 nm long-pass filter for device-30% UCNPs and pristine devices. (**d**) Quantum efficiency (IPCE) spectra of device-30% UCNPs and pristine devices.

The results, presented in Table 1, indicate that the lithium-based UCNPs enhanced the photovoltaics performance of the PSCs through optical and electrical effects. The introduction of lithium-based UCNPs into PSCs remarkably improved the harvesting of sunlight, and thus increased the photocurrent, while lithium doping within the mesoporous layer of the PSCs induced faster charge transport and improved the open circuit voltage, fill factor, and PCE values. Figure 4a and Table 1 display that device-30% UCNPs demonstrated the highest short circuit current density (J_SC_) and PCE, with a 4% enhancement in Jsc and a 13% enhancement in PCE in comparison to the pristine device, while the open circuit voltage (Voc) increased as the UCNPs increased. The enhancement in the photovoltaics performance of device-30% UCNPs could be attributed to the greater number of NIR photons converted by the UCNPs in the mesoporous layer to absorbed visible light photons by the perovskite light-harvesting layer, and therefore, converted directly into an additional photocurrent. In addition, Li-doping in the UCNPs host crystal improved the surface passivation (TiO_2_/Perovskite interface), which enabled a faster electron transport within the mesoporous layer of the PSCs cells. These results in enhanced short circuit current density (J_SC_), power conversion efficiency (PCE), and higher Voc of the fabricated PSCs devices, were in a good agreement with a previous study reported in [10]. The fill factor (FF) also showed a maximum value of 82.1 for device-30% UCNPs, as shown in Table 1. The excellent improvement in the FF (from 71.3% to 82.1%) was not only due to light harvesting by UCNPs, but also because the lithium dopant decreased the number of deep traps, which acted as recombination centers and induced faster charge transport within the TiO_2_, improving the open circuit voltage and fill factor, respectively [10].

Table 1 and Figure 4a,b show that increasing the mixing amount of UCNPs in the mesoporous layer of the fabricated devices results in lower J_SC_ and PCE. The decrease in the photovoltaic performance of device-40% UCNPs and device-50% UCNPs could be attributed to an excessive light back-scattering to the reflection of a large portion of the incident light out of the cell, resulting in a weakened absorption. Furthermore, a higher addition of the UCNPs resulted in a poor conductivity in the electron transport layer, as concluded from the reduction in the fill factor values, as shown in Table 1.

The NIR light-harvesting contribution to the photovoltaic performance of the device-30% UCNPs and control (pristine device) was investigated under AM 1.5 G standard sunlight with an 800 nm long-pass filter. Under only NIR illumination, device-30% UCNPs demonstrated a relatively higher performance in comparison to the pristine device, as shown in Figure 4c. This observation might be due to the optical upconversion luminescence of the UCNPs, in which the low-energy of the NIR photons of the incident light was converted to the high-energy of visible light photons by UCNPs and absorbed by the perovskite light-harvesting layer, resulting in an additional photocurrent. It was also important to test the quantum efficiency of the best fabricated device (device-30% UCNPs) in this work versus the pristine device. The measured quantum efficiency is the ratio of the number of carriers collected by the solar cell to the number of incident photons, limited by the band gap of the perovskite, which is about 1.5 eV. As demonstrated in Figure 4d, device-30% UCNPs showed a higher incident-photon-to-current conversion efficiency (IPCE) spectra in the region of 300–800 nm in comparison to the pristine device. This enhancement in the IPCE curve implied a better capability of the charge carriers’ collection and a lower charge recombination for device-30% UCNPs than those for the pristine device. The (IPCE) spectra of all fabricated devices were measured as illustrated in Figure S1. Even though a long-term stability study of the fabricated devices was not performed in this work, it is worth mentioning some possible problems associated with sunlight induced aging of the considered materials. According to previous studies, humidity and UV radiation from sunlight induce a catalytic reaction of TiO_2_ which leads to the decomposition of perovskite film [48,49].

## 4. Conclusions

In this work, we successfully synthesized a hybrid system combining two promising nanomaterials known to enhance the PCE of PSCs. High quality lithium-based UCNPs were synthesized, characterized, and then optimally mixed within the mesoporous layer of the PSCs. At an optimal mixing ratio of the synthesized UCNPs, PSCs (doped with 30% of UCNPs) demonstrated a higher J_SC_ and PCE than the undoped PSCs cells. Our results show that PSC based on the lithium-based UCNPs doped within the mesoporous layer achieved a high PCE of 19%, while the pristine device based only on the TiO_2_ mesoporous layer gave an efficiency of 16.5%. The reported results indicate that lithium-based UCNPs are a promising hybrid system for high performance PSCs.

## Figures and Tables

**Table 1:** Photovoltaic parameters of the fabricated devices.

Sample	Jsc (mA/cm^2^)	FF (%)	Voc (V)	PCE (%)
Pristine	21.49	71.3	1.084	16.5
Device with 15% UCNPs	21.85	72.7	1.112	17.64
Device with 30% UCNPs	22.34	82.1	1.013	18.6
Device with 40% UCNPs	21.73	77.1	1.082	18.12
Device with 50% UCNPs	21.49	76.8	1.01	16.99

## Data Availability

The data presented in this study are available on request from the corresponding author.

## Supplementary Materials

The following are available online at https://www.mdpi.com/article/10.3390/nano11112909/s1, Figure S1: The (IPCE) spectra of all fabricated devices in the region of 300–800 nm in comparison to the pristine device.
