# Supplementary material for: Lithium-Based Upconversion Nanoparticles for High Performance Perovskite Solar Cells
Source: Nanomaterials (Basel). 2021 Oct 30;11(11):2909. doi: 10.3390/nano11112909 (PMC8623711; doi:10.3390/nano11112909)
Supplement: Supplementary file 1 [file nanomaterials-11-02909-s001.zip › nanomaterials-1447995-supplementary.pdf]

Supplementary Materials

Lithium-Based Upconversion Nanoparticles for High Performance Perovskite Solar Cells

Masfer Alkahtani [1,6,*], Anas Ali Almuqhim [1], Hussam Qasem [1], Najla Alsofyani [1], Anfal Alfahd [1], Sultan M. Alenzi [2], Abdulaziz Aljuwayr [1], Yahya A. Alzahrani [2,3], Abdurahman Al-Badri [2], Mohammad Hayal Alotaibi [3], Abdulaziz Bagabas [3], Abdulaziz N. AlHazaa [4,5] and Philip R. Hemmer [6,7,8]

1 National Center for Renewable Energy, King Abdulaziz city for science and technology (KACST), Riyadh 11442, Saudi Arabia; amukhem@kacst.edu.sa (A.A.A.); hqasem@kacst.edu.sa (H.Q.); nalsofyani@kacst.edu.sa (N.A.); aalfahd@kacst.edu.sa (A.A.)

2 National Center for Nanotechnology and Semiconductors, King Abdulaziz City for Science and Technology (KACST), Riyadh 11442, Saudi Arabia; sultan0064@gmail.com (S.M.A.); yalzhrani@kacst.edu.sa (Y.A.A.); aalbadri@kacst.edu.sa (A.A.-B.)

3 National Petrochemical Technology Center (NPTC), Materials Science Research Institute (MSRI), King Abdulaziz City for Science and Technology (KACST), Riyadh 11442, Saudi Arabia; mhhalotaibi@kacst.edu.sa (M.H.A.); abagabas@hotmail.com (A.B.)

4 Research Chair for Tribology, Surface, and Interface Sciences (TSIS), Department of Physics and Astronomy, College of Science, King Saud University, Riyadh 11451, Saudi Arabia; aalhazaa@ksu.edu.sa

5 King Abdullah Institute for Nanotechnology, King Saud University, Riyadh 11451, Saudi Arabia

6 Institute for Quantum Science and Engineering, Texas A&M University, College Station, TX 77843, USA; prhemmer@exchange.tamu.edu

7 Department of Electrical and Computer Engineering, Texas A&M University, College Station, TX 77843, USA

8 FRC Kazan Scientific Center of RAS, Zavoisky Physical-Technical Institute, Sibirsky Tract, 10/7, 420029 Kazan, Russia

* Correspondence: mqhtani@kacst.edu.sa; Tel.: +966-553-322-891

1. incident-photon-to-current conversion efficiency (IPCE) measurements for all fabricated devices:

It was important to evaluate the (IPCE) performance of all fabricated devices in the region of 300–800 nm in comparison to the pristine device. Figure S1 demonstrates the IPCE spectra for all devices, where the external quantum efficiency (EQE) values started to increase to its maxima at the optimal doping level of the UCNPs in device-30% UCNPs. The IPCE spectra for higher mixing ratio of the UCNPs resulted in a lower capability of charge carriers' collection, a lower charge recombination, and low quantum efficiency values.

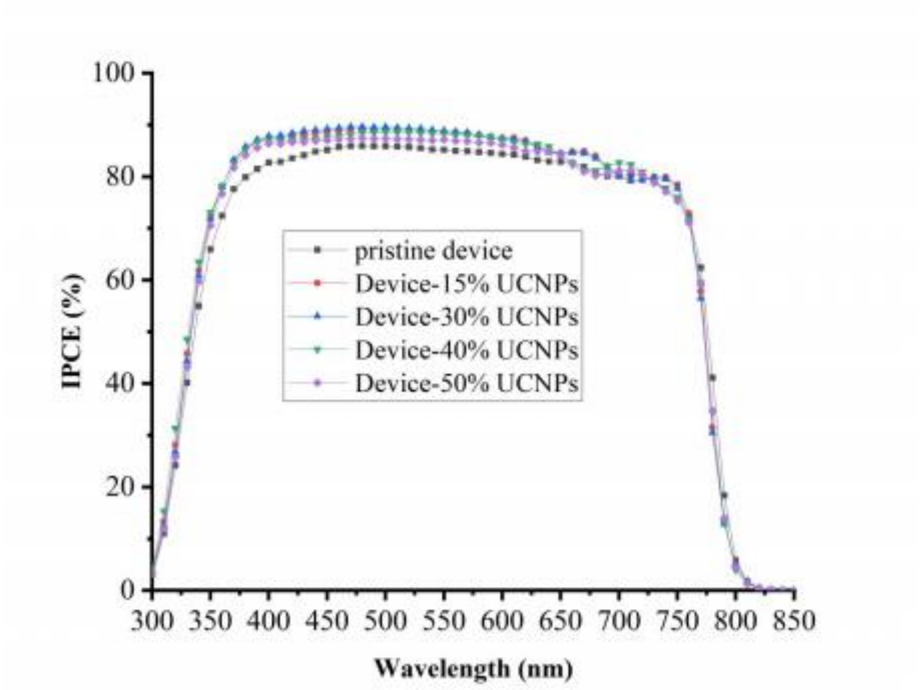

Figure S1. The (IPCE) spectra of all fabricated devices in the region of 300–800 nm to the pristine device.
